# A simple method for repeated in vivo sperm collection from laboratory mice

**DOI:** 10.1007/s10815-024-03201-x

**Published:** 2024-07-17

**Authors:** Sophie M. Burgstaller, Kerstin E. Auer, Thomas Rülicke

**Affiliations:** 1 Department of Biomedical Sciences and Pathobiology, University of Veterinary Medicine Vienna, Vienna, Austria; 2 Ludwig Boltzmann Institute for Hematology and Oncology, Vienna, Austria

**Keywords:** Mice, Ejaculation, In vivo sperm collection, Spermatozoa, Reproductive behavior

## Abstract

**Purpose:**

Mouse spermatozoa for archiving laboratory mice or for in vitro fertilization (IVF) are routinely obtained from the *cauda epididymis* of adult males sacrificed for this purpose. To avoid the death of the donor, we tested whether a precisely timed interruption of the mating act could be used for repeated sperm collection from laboratory mice.

**Methods:**

Sperm donors (B6D2F1) were mated with a receptive female, and mating behavior was observed. The stud was separated from the female 1–2 s after the onset of the ejaculatory shudder. The ejected copulatory plug with the yellowish viscous ejaculate was carefully removed from the penile cup.

**Results:**

A total of 80 ejaculates were successfully obtained from 100 ejaculations. The latency to first mount was 1.1 ± 1.1 min (mean ± SD) and to ejaculation 8.1 ± 4.7 min. The average number of mounts to ejaculation was 10.5 ± 5.8, and the mean number of spermatozoa per collected ejaculate was 1.86 ± 1.05 × 10^6^. An average fertilization rate of 76% was observed after IVF.

**Conclusions:**

Separating the stud from the female just before ejaculation is feasible, easy to learn, and requires no special equipment. The sperm count of collected ejaculates is lower than natural ejaculations, but higher than previous in vivo sperm collection methods achieved. We recommend this simple sperm collection method in mice, especially when the donor cannot be sacrificed and/or repeated sperm collection from the same animal is required for experimental purposes.

**Supplementary Information:**

The online version contains supplementary material available at 10.1007/s10815-024-03201-x.

## Introduction

In biomedical research, animal models are an important tool for studying physiological and pathophysiological processes in the context of a complex organism. Since the first reports on the production of transgenic animals using retroviral vectors [1] and the pronuclear injection of DNA constructs [2], the use of laboratory mice in basic and applied research has been steadily increasing. Genome engineering methods have allowed for the first time to generate specific mouse models to study genetic changes as the cause of abnormal processes underlying human and animal diseases. The development of other techniques to generate mouse models, such as gene targeting in embryonic stem cells [3] and endonuclease-mediated mutations [4, 5] has opened up further and more precise possibilities for targeted interventions in the mouse genome. Today, such animal models also play an important role in the development of therapies for rare human diseases, the exact causes of which are still poorly understood due to their rarity.

The number of genetically modified mouse models has long since outstripped the approximately 450 classical laboratory mouse strains described. The work of the International Knockout Mouse Consortium (IKMC) alone has generated a collection of genetically modified embryonic stem cells that allows studying the function and regulation of most of the approximately 20,000 protein-coding genes in separate mouse models [6]. In addition, CRISPR/Cas9 technology has provided a new, highly efficient way of altering the genomes of mice and other laboratory rodents [7]. As the number of mouse models continues to grow rapidly, their cost-effective archiving is particularly important, since it is impossible to maintain all lines permanently as breeding stock.

The successful archiving of mouse models using cryopreservation of pre-implantation embryos is based on the pioneering work of Whittingham, Leibo, and Mazur [8] and quickly became routine in many laboratories and repositories. Despite intensive efforts to preserve mouse lines using sperm, it was not until 20 years later that a method with reproducible results was established for the mouse [9]. However, several developments were needed both in cryopreservation and in vitro fertilization (IVF) with frozen mouse sperm before sperm freezing could be used routinely as a safe alternative to embryo freezing [10].

Mouse sperm for archiving is usually collected post-mortem from an isolated *cauda epididymis*. The quantity and quality of sperm obtained with this approach are high and usually require only two or three males to provide enough material to reliably cryopreserve a line. Even though the animal toll is low, the sacrificed males are no longer available for breeding or other purposes. For certain lines, even a small reduction in available animals can become problematic and lead to the loss of the line. For this reason, alternative, non-terminal methods for sperm collection have been developed and tested. In addition, repeated sperm samples from the same donor may also be of interest for the study of reproductive physiology and other scientific purposes, e.g., to study changes in sperm production and quality over time.

One way to save the life of the donor male is to remove the *cauda epididymis* unilaterally. However, the procedure is stressful for the animal and can only be performed once. Electroejaculation, used as an alternative in other mammalian species, also proved unsatisfactory in laboratory mice. It is stressful and even painful for the animals, and up to 22% of treated animals died as a result of the procedure [11, 12].

Another method described for obtaining mouse sperm is to rinse both uterine horns removed from freshly mated females. Although sperm obtained in this way showed reduced motility and vitality, it could be successfully used for IVF and cryopreservation [13, 14]. To prevent killing the female and also to avoid unwanted pregnancies, uterine lavage after prior cauterization of both fallopian tubes has been performed. This required the females to be anesthetized and the vaginal plug to be removed, which in some cases resulted in injury to the vagina. In addition, due to the relatively short fertilization time of mouse sperm, it is essential to flush the uterine horns after mating as soon as possible to obtain sufficiently motile sperm [15].

Microsurgical epididymal sperm aspiration (MESA) or percutaneous epididymal sperm aspiration (PESA) is another way to repeatedly collect sperm from the *cauda epididymis* of laboratory mice without killing the donors [16, 17]. Males only need to be briefly anesthetized for the puncture and can be returned to breeding once recovered. Although the quantity and quality of sperm obtained by aspiration are sufficient for IVF, it is usually insufficient for cryopreservation. In addition, the sperm quality after PESA is diminished, likely caused by the suction pressure during puncture and aspiration with a 30G injection needle [18]. A modification of MESA is therefore the collection of exuding sperm with a pipette directly from the surface of the punctured *cauda epididymis *in situ [19].

In summary, all currently existing methods for obtaining sperm from mice without killing the donor are stressful or invasive for the animals. They are also technically difficult to standardize, which limits the reproducibility of the results. While investigating the ejaculatory reflex as a stimulus for the formation of functional *corpora lutea*, McGill and Coughlin [20] found that male mice ejaculate even if they are separated from the female immediately after the onset of the ejaculatory shudder. As an alternative to previously described and practiced methods of in vivo sperm collection, we therefore tested whether a precisely timed interruption of the mating act could be used as a routine method for repeated and gentle collection of sperm from laboratory mice.

## Materials and methods

A total of 10 male and 20 female B6D2 (C57BL/6NCrl × DBA/2NCrl) F1 hybrids purchased from Charles River Laboratories (Germany) at 10 weeks of age were used to establish and evaluate the sperm collection method. Additionally, eight B6D2F1 females at 3–4 weeks of age were used as oocyte donors for IVF with the collected sperm. Hybrid mice were selected for their high fitness, sexual motivation, copulatory performance, and prominent copulatory plugs. Upon arrival, the mice were housed in type III Makrolon® cages (Tecniplast, Buguggiate, Italy) in a non-barrier rodent facility under controlled temperature (20 ± 1 °C) and relative humidity of 55 ± 10% with a 12:12 h light/dark cycle (lights on at 6:00 am). Males were housed individually. Females were housed in pairs instead of larger groups to avoid the spontaneous extra-coital pseudopregnancy, resulting in prolongation or suppression of the estrous cycle (Lee-Boot effect [21]). Food (ssniff® M-Z extrudate) and tap water were provided ad libitum. Cages were equipped with wooden bedding (LIGNOCEL® 3–4 S, Rettenmaier und Söhne GmbH, Rosenberg, Germany), nesting material (Pur-Zellin; Paul Hartmann GmbH, Wiener Neudorf, Austria), and cardboard tubes and houses (Special Diet Service, Claus GmbH, Limburgerhof, Germany) as enrichment. The animals were given 1 week of habituation before the start of the experiments.

### Sperm collection

To evaluate the sperm collection procedure, the following parameters were recorded: (i) latency from the introduction of a receptive female into the male’s cage to first mount, (ii) latency from the introduction of the female to ejaculation, (iii) number of mounts to ejaculation, and (iv) number and motility of spermatozoa collected per ejaculate. We further examined pregnancy rates in females who were used as mating partners.

All sperm collections were performed by the same person and in the same way (see supplementary video). The person performing the procedure was experienced in handling mice, but not in the tested procedure for obtaining ejaculates from mice. All ejaculations are included in the analysis. Males were mated repeatedly over a 7-week period, until each subject had ejaculated ten times, regardless of whether sperm collection was successful or not. We required 23 test days to achieve ten ejaculations from each of the ten males. After ejaculation, the male mice were given a rest period of at least 48 h before they were mated again.

Mating for sperm collection took place at the beginning of the light period at 06:00 a.m. until 11:00 a.m. at the latest. Ovulation time in mice is assumed to be in the first half of the dark phase, i.e., until around midnight. If this was the case, the presentation of the female occurred in the post-ovulatory phase, in which both the copulation rate and the fertilization rate are as good as in the ovulation phase [22]. To avoid distracting males in an unfamiliar environment, the female was always placed in the male’s cage.

We assessed a female’s readiness to mate by her reaction towards the male’s attempt to approach. Defensive behavior such as rearing up, kicking, or turning towards the male tended to indicate a lack of willingness to mate. Females in estrus, however, were usually quickly mounted by the male. Females that were not ready to mate were replaced one by one until a receptive female was found, which was then used as a partner for consecutive matings, given that ejaculation had been successfully interrupted.

Copulatory behavior, which includes thrusts and mounts, was closely monitored and as soon as intromission occurred, the male’s tail was grasped with one hand and the female’s tail with the other in order to separate the animals at the right time. Separation has to occur 1–2 s after the onset of ejaculatory shudder [20], which marks the onset of the ejaculatory reflex but not the onset of ejaculation. The animals were then gently separated by pulling them apart. It is important to pull on both tails at the same time, as the male holds the female with his legs during ejaculation (see supplementary video).

**Fig. 1:**
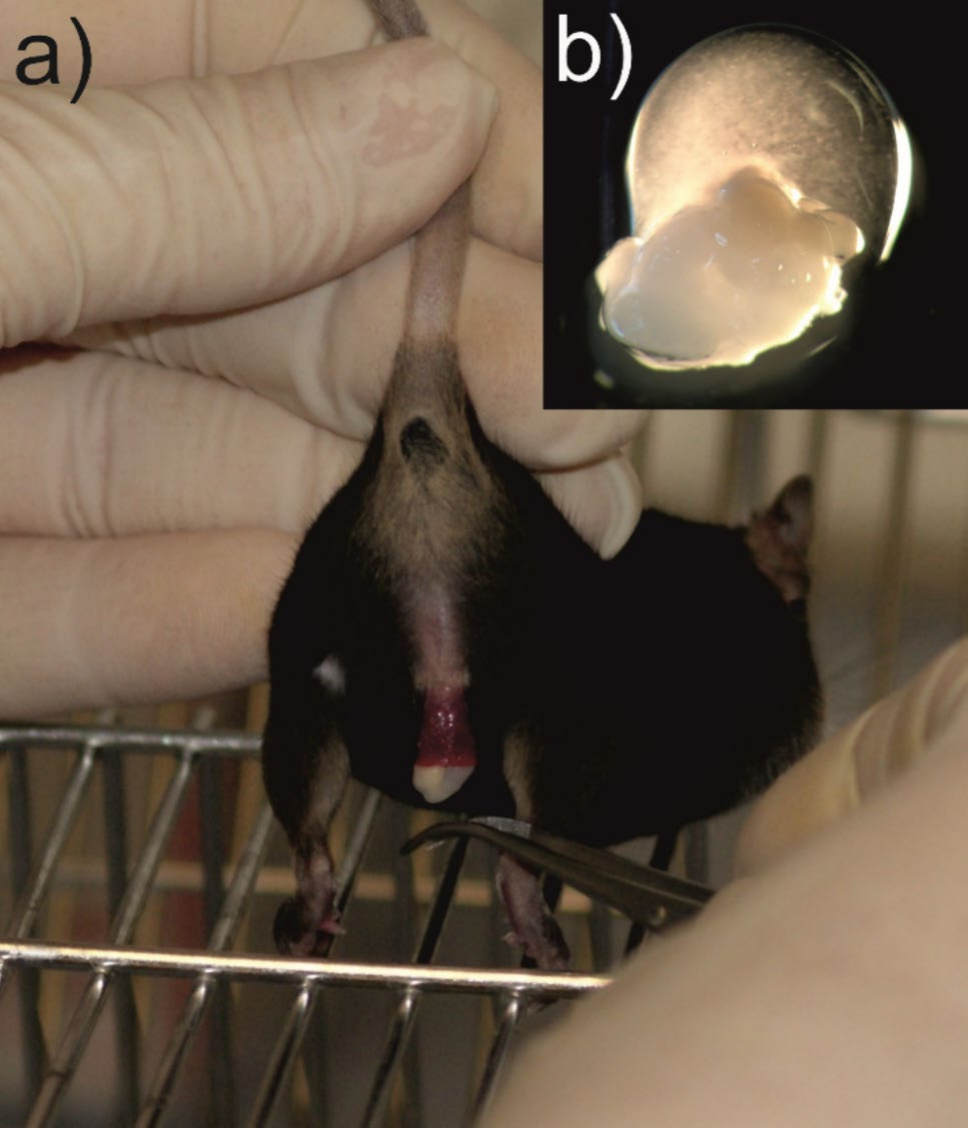
**a** Picture of the copulatory plug and the ejaculate of a male B6D2F1 mouse immediately after the separation from the female. After separation, the ejaculating male was placed with his front and hind legs on a cage lid. A curved forceps was used to remove the still elastic plug from the penile cup. **b** Mating plug with attached ejaculate in a drop of medium covered with mineral oil for sperm collection

After separation, the female was left in the cage while the ejaculating male was placed on a cage lid. To prevent the male from grasping the erect penis with his hind legs and injuring it with his claws, he is required to hold on to the bars of the cage lid with his front and hind legs (Fig. 1a). The shape of the penis visibly changes at the distal end into a “penile cup” and appears in a dark red color. This transformation is immediately followed by the ejaculation of the sperm-rich fluid, directly followed by the copulatory plug. Using a curved forceps, the still elastic plug can then be carefully removed from the penile cup. The ejaculate is clearly visible as a yellowish viscous drop on the white plug (see supplementary video). The plug with the ejaculate was briefly placed in a 100 µl drop of human tubal fluid (HTF) medium (for the detailed composition of HTF, see [17]) (Fig. 1b) covered with mineral oil (Sigma M5310). Under a binuclear microscope, the ejaculate was then separated from the plug by carefully scraping the plug off with a pair of watchmaker forceps. Due to the uneven surface of the plug, the ejaculate may not be fully extracted.

After removing the copulatory plug from the petri dish, the sperm suspension was incubated for 20 min at 37 °C in a humidified atmosphere of 5% CO_2_ in the air before sperm motility and quantity was analyzed.

Immediately after sperm collection, the mated female was examined for a copulatory plug. If the separation from the stud was timed properly, no plug formed in the vagina, and the female could be used again as a mating partner for another male. After being used in the experiment, females were housed individually. Female body weight was measured after sperm collection and 12 days later to diagnose unwanted pregnancy by a significant increase in body weight. If pregnant, females were left to give birth and were re-used as mating partners during post-partum estrus. Since induced pseudopregnancy would have been terminated 12 days after mating due to the absence of placental lactogens required to stimulate the *corpora lutea* in the second half of pregnancy [23], the non-pregnant females were subsequently used again as mating partners for sperm collection.

### Examination of sperm quality and quantity

Immediately after incubation, an aliquot of the sperm suspension was used to determine sperm motility and sperm number in the ejaculate by light microscopy at 200 × magnification and negative phase contrast. The percentage of motile sperm was assessed subjectively based on the experience of the experimenter. A sample of the sperm suspension was placed on a microscope slide and covered with a coverslip. Motile sperm were counted as those with progressive movement or at least clear flagellar movement. Non-motile spermatozoa included all spermatozoa without flagellar movement and sperm heads without flagella.

To determine the sperm count, a 10 µl aliquot was taken from the sperm suspension and depending on the visually determined sperm density diluted 5, 10, or 15 times with aqua bidest, before being loaded on a Neubauer cell counting chamber. The sperm count was measured twice for each sample, and the average was calculated.

### In vitro fertilization

To test the fertilization ability of sperm from the collected ejaculates, we performed IVF. Therefore, we collected one ejaculate from four randomly selected males and performed the IVF assays on two separate days (two males per day). Four juvenile females were each superovulated by intraperitoneal administration of 5 IU PMSG and 5 IU hCG (Folligon and Chorulon, both from Intervet, Vienna, Austria,) at 8 am, 48 h apart. The next morning, sperm was collected from two males by separating the mating mice after the onset of ejaculatory shudder as described. The copulatory plug with the ejaculate was placed in a drop of 90 µl of modified Krebs–Ringer bicarbonate medium (TYH, for detailed composition, see [17]) plus 0.75 mM methyl-β-cyclodextrin (MBCD: Sigma C4555) and containing 1.0 mg/mL polyvinyl alcohol (Sigma P8136) for preincubation [24]. The spermatozoa were then incubated at 37 °C for approximately 60 min for capacitation before use. During this incubation period, the oocyte donors were sacrificed by cervical dislocation. The oviducts were dissected and placed in mineral oil (Sigma M5310) covering a 200 µl drop of fertilization medium containing HTF plus 1.0 mM reduced glutathione (GSH: Sigma: G4251) [25]. One oviduct from each of the four donor females was assigned to each of the two males. The swollen ampullae were opened with watchmaker forceps and the four cumulus–oocyte complexes of two donor females were each drawn into one fertilization drop. For insemination, 5 µl of sperm suspension collected from the peripheral part of the preincubation drop (the zone of spermatozoa with higher motility) was added to the cumulus–oocyte complexes without prior determination of the sperm count. The fertilization was started between 08:30 and 09:30 in the morning. The fertilization dishes were then incubated for approximately 4 h at 37 °C under 5% CO_2_ in air to allow fertilization to occur. Morphologically intact, presumptive zygotes were washed and then cultured in HTF medium without GSH overnight. The next morning two-cell embryos were counted, and the two-cell rate was calculated as a percentage of the total number of oocytes in the IVF per sperm donor.

### Statistics

IBM SPSS Statistics (version 29) was used for descriptive statistics, statistical analysis, and data visualization including artwork. To compare mating behavior and sperm number between the first and second male to mate, we confirmed normal data distribution using the Kolmogorov–Smirnov test and homogeneity of variances using the Levene test, before applying independent-samples *T* tests.

## Results

**Fig. 2:**
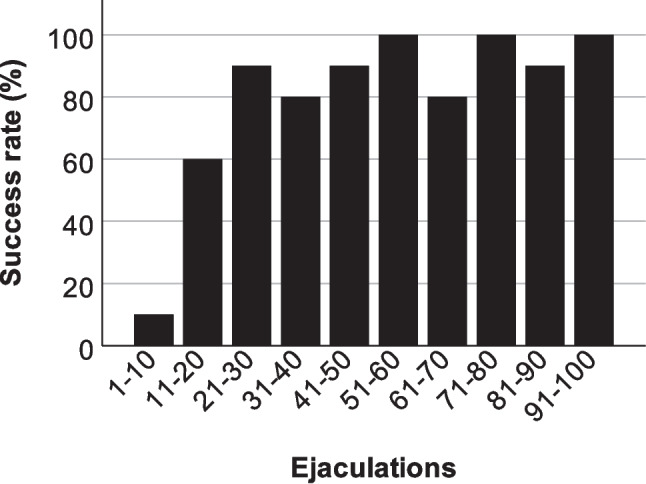
Percentage of successful sperm collection over the course of 100 ejaculations. Over a period of seven weeks, ten B6D2F1 hybrid males were mated repeatedly until each male had ejaculated ten times. In the first ten ejaculations, only one ejaculate was obtained; after the first 20 ejaculations, the success rate was consistently between 80 and 100%

As not every mating with a receptive female resulted in copulation and ejaculation, a minimum of 11 and a maximum of 14 matings were required to collect ten ejaculates per male. The successful sperm collection by separating females and males at the beginning of the ejaculatory shudder, which marks the initiation of the ejaculatory reflex, requires a certain amount of experience. A total of 80 ejaculates were successfully obtained from 100 ejaculations by mating with receptive females, revealing an average success rate of 80%. Importantly, success rates increased from 35% after the first 20 ejaculations to an average of 91.3% in the following 80 ejaculations (Fig. 2). It took nine test days to collect the first 20 ejaculations. The remaining 80 ejaculations were achieved in just 14 test days. The number of successfully obtained ejaculates varied between 6 and 10 out of 10 ejaculations per male (Supplementary Table 1).

Males initiated sexual behavior, such as chasing and sniffing, immediately after a receptive female was presented. Among all males and matings with ejaculations, the mean time to first mount was 1.1 ± 1.1 min (mean ± SD). In some cases, the first attempt to mount occured immediately after female introduction. The longest measured latency to the first mount was 8 min.

**Fig. 3:**
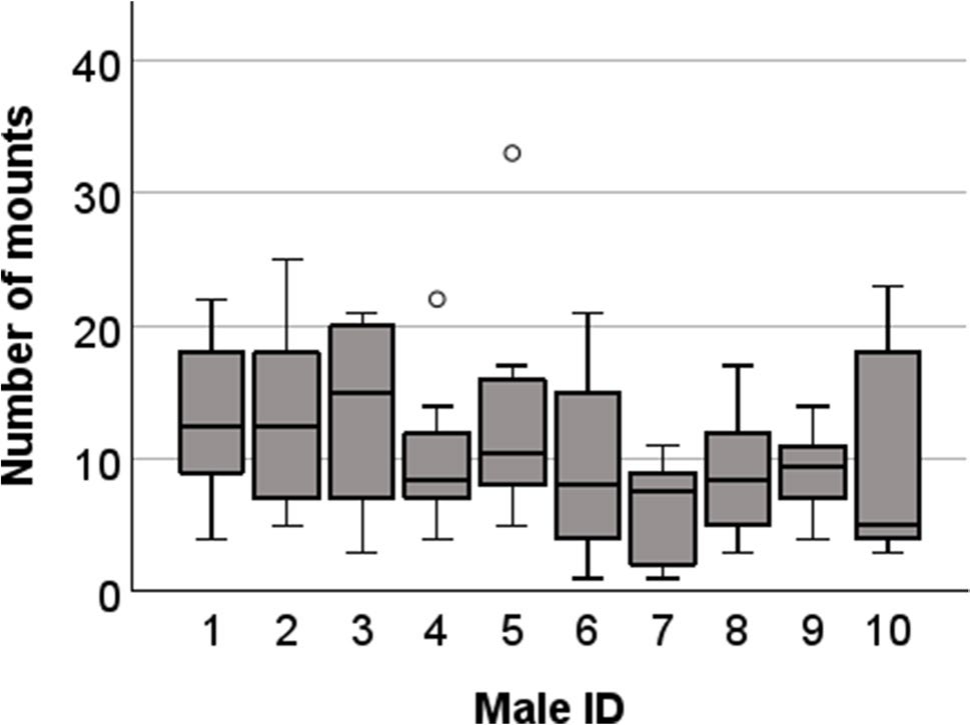
Boxplot of the number of mounts till ejaculation per male. Over a period of seven weeks, ten males were mated repeatedly until each had ejaculated ten times. The number of mounts before each ejaculation was counted. Circles (○) indicate mild outliers (Q3 + 1.5 × IQR)

Mating behavior in laboratory mice usually involves several mounts to intromission and ejaculation. The number of mounts observed before ejaculation varied considerably between males. In some matings, ejaculation occurred on the first mount. In other cases, more than 20 previous mounts were observed. Among all males and matings with ejaculations, the mean number of mounts to ejaculation was 10.5 ± 5.8 (Fig. 3).

**Fig. 4:**
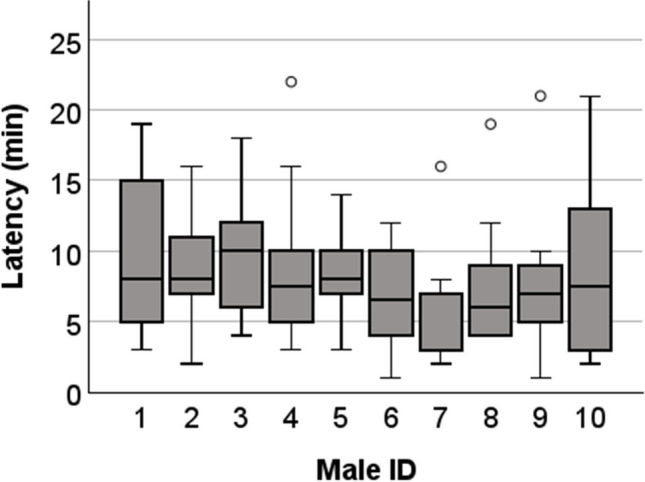
Boxplot of the latency till ejaculation per male. Over a period of seven weeks, males were mated repeatedly until each male had ejaculated ten times. The ejaculation latency from the introduction of the female was measured in minutes. Circles (○) indicate mild outliers (Q3 + 1.5 × IQR)

The number of mounts per minute was relatively constant at 1.75 ± 0.75, suggesting that the males mated continuously with the female until ejaculation. The latency to ejaculation was on average 8.1 ± 4.7 min (Fig. 4) (ranging from 1 to 21 min) and also varied with the number of mounts. The fewer mounts, the shorter the latency to ejaculation, and vice versa.

**Fig. 5:**
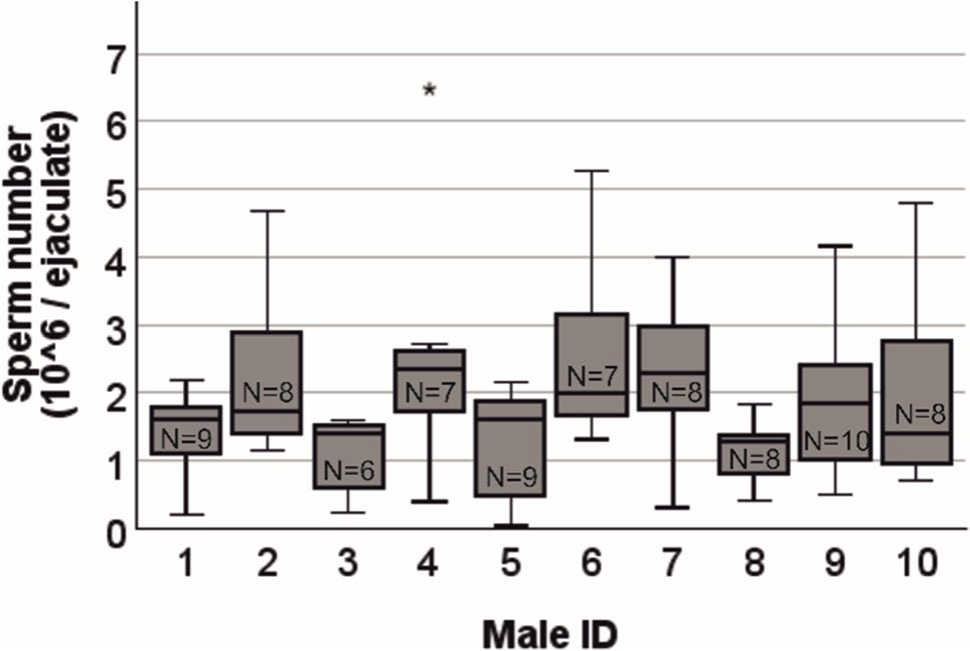
Boxplot of male sperm number (10^6^/ejaculate) in collected ejaculates per male. Over a period of seven weeks, males were mated repeatedly until each male had ejaculated ten times. A minimum of six and a maximum of ten ejaculates were successfully collected from males. Asterisks (*) indicate outliers (Q3 + 3 × IQR)

Collected ejaculates contained on average 1.86 ± 1.05 × 10^6^ spermatozoa, ranging from a minimum of 0.04 × 10^6^ to a maximum of 6.48 × 10^6^ (Fig. 5 and Supplementary Table 1).

**Fig. 6:**
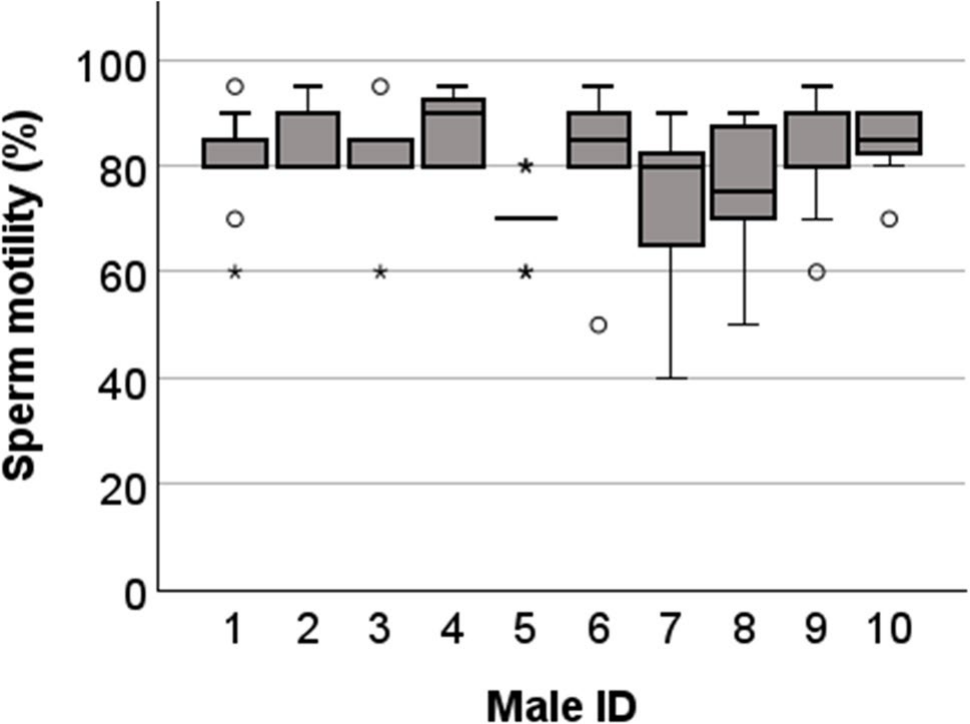
Boxplot of male sperm motility (%) in collected ejaculates per male. Over a period of seven weeks, males were mated repeatedly until each male had ejaculated ten times. Successfully collected ejaculates (*N* = 6–10/male) were analyzed. Circles (○) indicate mild outliers (Q1/Q3 ± 1.5 × IQR) and asterisks (*) indicate outliers (Q1/Q3 ± 3 × IQR)

The relative proportion of motile sperm per ejaculate varied between 50 and 95% (mean ± SD: 79.8 ± 10.5) (Fig. 6).

If mating partners were separated at the right time point, the female was used for mating with another male. On 13 occasions, it was possible to obtain an ejaculate from the first and second mating partners of the same female, and we investigated whether female mating status affected the mating behavior and collected sperm number of the second male to mate. The mean number of sperm per ejaculate did not differ between males (*t* = 0.925, *p* = 0.364) and was on average 1.87 ± 0.78 × 10^6^ for the first males to mate and 1.57 ± 0.83 × 10^6^ for the second males. Even though, males from the second mating took more mounts to ejaculate (mean ± SD: 10.15 ± 6.09 vs 8.00 ± 3.81), and their latency to ejaculate was longer (mean ± SD: 7.85 ± 4.18 vs. 5.31 ± 2.32 min) than males from the first mating, these differences were statistically not significant (mount number: *t* =  − 1.081, *p* = 0.291; ejaculation latency: *t* =  − 1.914, *p* = 0.068).

Importantly, we can confirm that the sperm collected by this method is fertile, as evident from the average fertilization rate of 76% in IVF trials (Table 1).

**Table 1:** Results of performed IVFs with sperm obtained from the ejaculates of four randomly selected donor males

Male	Number oocytes/two-cell stages	Two-cell rate %
1	66/60	90.9
2	95/51	53.7
3	53/51	96.2
4	52/39	75.0

The success of the method for obtaining sperm was also expressed in the number of induced or non-induced pregnancies in the female mating partners. While all but one of the females that were separated too late had a vaginal plug and became pregnant, none of the females that were separated at the right time had a vaginal plug or became pregnant.

## Discussion

For archiving the numerous mouse lines that are being constantly developed for biomedical research, freezing of sperm has become increasingly important compared to the archiving of pre-implantation embryos. In routine practice, sperm are obtained from the *cauda epididymis* of previously killed donor males. We have successfully tested a novel, non-terminal sperm collection method for mice by the separation of mating partners shortly before the onset of ejaculation. As sperm can be collected repeatedly with this approach, the method is also of interest for other scientific purposes. These include the assisted reproduction of a line by IVF or the assessment of the donor’s sperm quality over longer periods of time or at different stages of life. Currently, there is no other method to repeatedly collect sperm samples from mice without stressing the donors.

To obtain the ejaculate, the male has to be separated from the female within 1–2 s after the onset of the ejaculatory shudder. Recognizing the correct time point to separate and carrying out the separation can be learned quickly. It is worth noting that the success rate for the first ten ejaculations was only 10% but increased rapidly thereafter. After the first 20 ejaculations, the success rate in obtaining the ejaculate was on average over 90%. None of the females that were separated at the proper time and therefore had no vaginal plug became pregnant. If, however, separation would occur too late, the Bruce effect could be used to terminate the unwanted pregnancy by exposing the female to the pheromones of an unfamiliar male [26].

During the 11 to 14 matings per male, we did not observe a decrease in the males’ readiness to mate. This observation suggests that the separation just before ejaculation and the subsequent removal of the copulatory plug with the ejaculate from the penile cup does not inflict a stressful or otherwise adverse event for donor males.

The most time-consuming aspect of this sperm collection method was the search for a receptive female, as female behavior did not always clearly indicate acceptance or rejection of the male. On some scheduled test days, none of the used females could be identified as clearly receptive. Increasing the number of available females would have helped to overcome this problem, as we temporarily lost females from the pool due to pregnancies, especially at the beginning of the study. However, once found, an estrous female can be presented to several donor males in succession on the same day, given that the males are separated at the correct time point before ejaculation. Here, it was possible to obtain sperm from all ten males tested after mating with the same receptive female. Estrous females used also showed no decline in their readiness to mate with several males in succession, a behavior supported by observations of multiple mating and multiple paternity in *Mus domesticus* [27] and *Mus musculus* [28]. As an alternative to mating female mice, estrus cycle examination using vaginal swabs could be used to identify receptive females [29].

In an experiment with male offspring of pregnant wild-caught *Mus m. domesticus* females, the sperm count in mouse ejaculates was determined by flushing the reproductive tract of the female B6CF1 mating partners. The results showed a large variation between males, ranging from 3.48 × 10^6^ to 16.53 × 10^6^ sperm per recovered ejaculate [30]. The mean sperm count per ejaculate in our study was distinctly lower (1.86 × 10^6^). However, loss of sperm due to adherence to the forceps and the copulatory plug cannot be ruled out. Separation of the ejaculate from the plug can be tricky due to the uneven surface of the plug. We did not determine the volumes of the viscous ejaculates to avoid any negative effects on sperm quality due to aspiration. The lower number of spermatozoa and the large variation between the collected ejaculates may further suggest that the influence of the separation procedure immediately after the onset of the ejaculatory shudder reduces the amount of spermatozoa released in the male’s ejaculate. Alternatively, the B6D2F1 hybrid males used in our study might a priori have a lower sperm count than wild-caught *Mus m. domesticus*.

Another possible factor influencing the amount of released sperm could be the successive use of the same female mating partner. Once the sperm collection method was mastered in the present experiment, only one or two receptive females were required as a mating partner for the males on each day of the study. However, it has been reported that the mating behavior of males appears to be influenced by whether the female has been previously mated. The mating behavior of subsequent males was characterized by significantly more intromissions before ejaculation and a significantly longer latency to ejaculation [31]. Therefore, we wondered whether in our investigation a previously mated female could have an effect on the mating behavior and ejaculated sperm number of the subsequent male. In our study, we could not distinguish between mounts with and without intromission when observing mating behavior. However, both the number of mounts and the latency to ejaculation slightly increased if the female had previously mated with another stud male. Although these differences were statistically not significant and do not call the practicality of the semen collection method into question, they are in line with findings from previous research and are potentially interesting for reproductive biology.

For efficient archiving of mouse strains, a high yield of motile spermatozoa per donor male is advantageous. Using the standard method of obtaining mouse sperm from the *cauda epididymis*, the mean number of spermatozoa obtained from BALB/c males was 18.4 × 10^6^ [32], and 10.0 × 10^6^ for wild-caught male offspring from only one *cauda epididymis* [33]. In routine practice, the sperm collected from both *cauda epididymides* of a donor male is usually divided into ten freezing straws, each containing more than 1 × 10^6^ sperm in 10 µl of sperm suspension. Even taking into account the losses due to freezing, this is far more sperm per straw than is required for successful IVF [34]. The recommended concentration of motile sperm for IVF is 1 × 10^6^ to 2.5 × 10^6^ sperm/mL [35]. With the sperm retrieval method presented, more than one ejaculate would normally be required to safely archive a line, which is feasible over a short period of time. Due to the high variability in the number of spermatozoa of successfully collected ejaculates, as shown in Supplementary Table 1, it may be necessary to repeat the procedure with the same donor in order to safely archive a strain. However, sperm collection with the presented method can be repeated as long as the donor is reproductively active.

With an average number of 1.86 × 10^6^ sperm, the presented method achieves a greater sperm yield than electroejaculation. When different electroejaculation devices were tested, the total number of sperm per ejaculate varied between 0.05 × 10^6^ and 1.4 × 10^6^. In addition to the high donor mortality rate of up to 22%, a significant reduction in the motility and velocity of the retrieved sperm is another disadvantage of this method [12]. At 0.4 × 10^6^ to 0.7 × 10^6^, the amount of sperm obtained by puncturing the *cauda epididymis* was also significantly lower than that obtained by the introduced separation method [17, 19]. The use of a very small diameter cannula to aspirate sperm has potentially also negative effects on the quality of sperm [18]. Although repeated sperm retrieval is also possible using electroejaculation or epididymal puncture, it is important to note that both procedures can only be performed under anesthesia. Therefore, the significant stress of the donor animals has to be taken into consideration when evaluating these methods.

Collection of ejaculates from the reproductive tract of a freshly mated female provides a large sperm yield and could be repeated if necessary. However, this method requires the killing of the female. Sperm retrieved from the reproductive tract of previously mated females has been successfully used in IVF after cryopreservation [14]. The sperm from the ejaculates used in the here presented analysis were not previously cryopreserved, but their excellent fertilization ability was confirmed with an average of 76% of two-cell embryos after IVF (Table 1). In addition, the proportion of motile sperm in the collected ejaculates from all test males was usually at least 80%, which is a very good basis for cryopreservation (Fig. 6).

Several studies indicate qualitative differences between ejaculated mouse sperm and sperm obtained directly from the epididymis, which are mainly explained by the influence of bioactive substances in the seminal plasma. For example, in mouse ejaculates retrieved from the uteri of previously mated females, a greatly increased nuclease activity was detected in both spermatozoa and seminal plasma, which was much less pronounced in epididymal spermatozoa and epididymal fluid. This difference could lead to an increased susceptibility of ejaculated sperm to DNA damage under cryopreservation conditions, thus compromising the integrity of archived sperm [36]. Sperm retrieved from the uterus after ejaculation also differ from epididymal sperm in their swimming pattern and, most importantly, are more efficient at penetrating the cumulus-oocyte complex in vitro [37].

Capacitation of the sperm is a prerequisite for fertilization. A fundamental process of mammalian sperm capacitation is the efflux of cholesterol and phospholipids from the sperm membrane. For the maturation of epididymal spermatozoa, the TYH preincubation medium contains polyvinyl alcohol as a substitute for bovine serum albumin and β-cyclodextrins, which acts as cholesterol and phospholipid acceptors to induce and support the capacitation process [24, 38].

In contrast to epididymal spermatozoa, ejaculated spermatozoa are coated with seminal plasma proteins. The secretion of the mouse seminal vesicle is the major component of the seminal plasma and contains several seminal vesicle proteins (SVS1-7) that not only influence the formation of the copulatory plug but also control sperm fertility [39]. Using SVS2 knockout males, it has been shown that this seminal vesicle protein suppresses premature ectopic activation of sperm maturation in the uterus, thus protecting the integrity and survival of sperm in the female reproductive tract. As a result, almost all sperm from wild-type males still have an intact acrosome at the isthmus to the oviduct, and the acrosome reaction as the completion of the maturation process essentially only takes place in the ampulla of the oviduct. In the absence of SVS2, sperm begin to die in the uterine cavity, and their in vivo fertility is drastically reduced. In contrast, during IVF, epididymal spermatozoa from SVS2^−/−^ males did not differ from wild-type spermatozoa in terms of motility and in vitro fertilization rate [40].

Seminal vesicle excision in male mice resulted in a greatly reduced pregnancy rate, similar to mating with SVS2 knockout males, due to abnormal development of the pre-implantation embryonic stages. In addition, abnormalities in growth and other metabolic parameters were observed in the few offspring [41]. Therefore, seminal plasma factors appear not only to ensure the survival and fertility of ejaculated sperm, but also to influence the developmental conditions of the embryos during pregnancy and the phenotypic characteristics of the offspring.

Cryopreserved sperm is routinely used in IVF to revitalize archived mouse lines. Whether there is a qualitative difference between ejaculated and epididymal spermatozoa for this application cannot be deduced from the available test results. However, from the results discussed on the influence of seminal plasma on fertilization success, it can be concluded that ejaculated sperm would be preferable for artificial in vivo insemination.

In the present study, males and females of an F1 hybrid strain were used to exploit the heterosis effect on fertility and specifically on male libido. However, inbred and hybrid strains of laboratory mice can differ significantly in their sexual behavior. It has been shown that C57BL/6 J males, the most commonly used inbred strain for mouse genome modification, mate faster than BALB/c or DBA/2 J males [42, 43]. The introduced collection method of ejaculates must therefore be tested in practice for the strain or genetic background in question.

In summary, the separation of mating mice at the onset of the ejaculation reaction is a simple and quick-to-learn method for obtaining reasonable amounts of good-quality mouse sperm in vivo. Thus, the presented method can contribute to the 3Rs concept by reducing the number of animals required compared to the standard procedure using epididymal sperm from dissected *cauda epididymides*. More importantly, it contributes to refinement, as all other existing techniques for repeated sperm collection are invasive and stressful for the animals. Finally, this method can be used to collect sperm not only for IVF or for archiving lines, but also, for example, for studies that require repeated sperm samples from the same individual over time.

## Supplementary Information

Below is the link to the electronic supplementary material.

Supplementary file1 (DOCX 15 KB)

Supplementary file2 (MP4 131971 KB)

## References

[1] Jaenisch R. Germ line integration and Mendelian transmission of the exogenous Moloney leukemia virus. Proc Natl Acad Sci U S A. 1976;73(4):1260–4. 1063407 10.1073/pnas.73.4.1260 PMC430242

[2] Gordon JW, Ruddle FH. Integration and stable germ line transmission of genes injected into mouse pronuclei. Science. 1981;214(4526):1244–6. 6272397 10.1126/science.6272397

[3] Thomas KR, Capecchi MR. Site-directed mutagenesis by gene targeting in mouse embryo-derived stem cells. Cell. 1987;51(3):503–12. 2822260 10.1016/0092-8674(87)90646-5

[4] Sung YH, Baek IJ, Seong JK, Kim JS, Lee HW. Mouse genetics: catalogue and scissors. BMB Rep. 2012;45(12):686–92. 23261053 10.5483/BMBRep.2012.45.12.242 PMC4133813

[5] Wang H, Yang H, Shivalila CS, Dawlaty MM, Cheng AW, Zhang F, Jaenisch R. One-step generation of mice carrying mutations in multiple genes by CRISPR/Cas-mediated genome engineering. Cell. 2013;153(4):910–8. 23643243 10.1016/j.cell.2013.04.025 PMC3969854

[6] Ringwald M, Iyer V, Mason JC, Stone KR, Tadepally HD, Kadin JA, Bult CJ, Eppig JT, Oakley DJ, Briois S, et al. The IKMC web portal: a central point of entry to data and resources from the International Knockout Mouse Consortium. Nucleic Acids Res. 2011;39:849–55. 10.1093/nar/gkq879 PMC3013768 20929875

[7] Schick JA, Seisenberger C, Beig J, Burger A, Iyer V, Maier V, Perera S, Rosen B, Skarnes WC, Wurst W. CRISPR-Cas9 enables conditional mutagenesis of challenging loci. Sci Rep. 2016;6:32326. 27580957 10.1038/srep32326 PMC5007477

[8] Whittingham DG, Leibo SP, Mazur P. Survival of mouse embryos frozen to -196 degrees and -269 degrees C. Science. 1972;178(4059):411–4. 5077328 10.1126/science.178.4059.411

[9] Takeshima T, Nakagata N, Ogawa S. Cryopreservation of mouse spermatozoa. Jikken Dobutsu. 1991;40(4):493–7. 1748166 10.1538/expanim1978.40.4_493

[10] Sztein JM, Takeo T, Nakagata N. History of cryobiology, with special emphasis in evolution of mouse sperm cryopreservation. Cryobiology. 2018;82:57–63. 29660317 10.1016/j.cryobiol.2018.04.008

[11] Snyder RL. Collection of mouse semen by electroejaculation. Anat Rec. 1966;155(1):11–4. 6006797 10.1002/ar.1091550103

[12] Tecirlioglu RT, Hayes ES, Trounson AO. Semen collection from mice: electroejaculation. Reprod Fertil Dev. 2002;14(5–6):363–71. 12467362 10.1071/RD02015

[13] Whittingham DG. Fertilization of mouse eggs in vitro. Nature. 1968;220(5167):592–3. 5686738 10.1038/220592a0

[14] Songsasen N, Leibo SP. Live mice from cryopreserved embryos derived in vitro with cryopreserved ejaculated spermatozoa. Lab Anim Sci. 1998;48(3):275–81. 10090028

[15] Foxworth WB, Carpenter E, Kraemer DC, Kier AB. Nonsurgical and nonlethal retrieval of mouse spermatozoa. Lab Anim Sci. 1996;46(3):352–4. 8799947

[16] Del Val GM, Robledano PM. In vivo serial sampling of epididymal sperm in mice. Lab Anim. 2013;47(3):168–74. 23760960 10.1177/0023677213478411

[17] Boersma A, Olszanska O, Walter I, Rulicke T. Microsurgical and percutaneous epididymal sperm aspiration for sperm collection from live mice. J Am Assoc Lab Anim Sci. 2015;54(5):471–7. 26424244 PMC4587614

[18] Windhofer L, Boersma A, Dahlhoff M, Rulicke T, Auer KE 2023 The impact of percutaneous epididymal sperm aspiration on sperm quality in mice. Reprod Fertil 4(2). 10.1530/RAF-23-0017 PMC10305637 37227214

[19] Moreno-Del Val G, Munoz-Robledano P, Caler AJ, Morante J. A method for multiple sampling mouse sperm in vivodagger. Biol Reprod. 2023;108(2):197–203. 36308433 10.1093/biolre/ioac194

[20] McGill TE, Coughlin RC. Ejaculatory reflex and luteal activity induction in Mus musculus. J Reprod Fertil. 1970;21(2):215–20. 5462643 10.1530/jrf.0.0210215

[21] Van Der Lee S, Boot LM. Spontaneous pseudopregnancy in mice. Acta Physiol Pharmacol Neerl. 1955;4(3):442–4. 13301816

[22] Nakao S, Ito K, Sugahara C, Watanabe H, Kondoh G, Nakagata N, Takeo T. Synchronization of the ovulation and copulation timings increased the number of in vivo fertilized oocytes in superovulated female mice. PLoS ONE. 2023;18(2):e0281330. 36745586 10.1371/journal.pone.0281330 PMC9901804

[23] Thordarson G, Galosy S, Gudmundsson GO, Newcomer B, Sridaran R, Talamantes F. Interaction of mouse placental lactogens and androgens in regulating progesterone release in cultured mouse luteal cells. Endocrinology. 1997;138(8):3236–41. 9231773 10.1210/endo.138.8.5309

[24] Takeo T, Hoshii T, Kondo Y, Toyodome H, Arima H, Yamamura K, Irie T, Nakagata N. Methyl-beta-cyclodextrin improves fertilizing ability of C57BL/6 mouse sperm after freezing and thawing by facilitating cholesterol efflux from the cells. Biol Reprod. 2008;78(3):546–51. 18046011 10.1095/biolreprod.107.065359

[25] Takeo T, Nakagata N. Reduced glutathione enhances fertility of frozen/thawed C57BL/6 mouse sperm after exposure to methyl-beta-cyclodextrin. Biol Reprod. 2011;85(5):1066–72. 21778138 10.1095/biolreprod.111.092536

[26] Bruce HM. An exteroceptive block to pregnancy in the mouse. Nature. 1959;184:105. 13805128 10.1038/184105a0

[27] Dean MD, Ardlie KG, Nachman MW. The frequency of multiple paternity suggests that sperm competition is common in house mice (Mus domesticus). Mol Ecol. 2006;15(13):4141–51. 17054508 10.1111/j.1365-294X.2006.03068.x PMC2904556

[28] Thonhauser KE, Raveh S, Hettyey A, Beissmann H, Penn DJ. Why do female mice mate with multiple males? Behav Ecol Sociobiol. 2013;67(12):1961–70. 24273373 10.1007/s00265-013-1604-8 PMC3827896

[29] Byers SL, Wiles MV, Dunn SL, Taft RA. Mouse estrous cycle identification tool and images. PLoS ONE. 2012;7(4):e35538. 22514749 10.1371/journal.pone.0035538 PMC3325956

[30] Ramm SA, Stockley P. Ejaculate allocation under varying sperm competition risk in the house mouse Mus musculus domesticus. Behav Ecol. 2007;18:491–5. 10.1093/beheco/arm003

[31] Ramm SA, Stockley P. Sequential male mate choice under sperm competition risk. Behav Ecol. 2014;25(3):660–7. 24822023 10.1093/beheco/aru037 PMC4014308

[32] Zavos PM, Correa JR, Zarmakoupis PN. Epididymal spermatozoa: recovery and subsequent improvements of mouse epididymal spermatozoa via the SpermPrep filtration method. Tohoku J Exp Med. 1995;175(2):101–9. 7541168 10.1620/tjem.175.101

[33] Bayram HL, Franco C, Brownridge P, Claydon AJ, Koch N, Hurst JL, Beynon RJ, Stockley P. Social status and ejaculate composition in the house mouse. Philos Trans R Soc Lond B Biol Sci. 1813;2020(375):20200083. 10.1098/rstb.2020.0083 PMC7661446 33070725

[34] Pedersen HS, Liu Y, Foldager L, Callesen H, Larsen K, Sorensen MT. Calibration of sperm concentration for in vitro fertilization in a mouse reprotoxicity model. Toxicol In Vitro. 2019;55:58–61. 30476541 10.1016/j.tiv.2018.11.009

[35] Nagy A, Gerstenstein M, Vintersten K, Behringer R: Assisted reproduction. In: *Manipulating the mouse embryo.* 3rd ed. edn: Cold Spring Harbor Laboratory Press; 2003: 565–579.

[36] Yamauchi Y, Ajduk A, Riel JM, Ward MA. Ejaculated and epididymal mouse spermatozoa are different in their susceptibility to nuclease-dependent DNA damage and in their nuclease activity. Biol Reprod. 2007;77(4):636–47. 17596560 10.1095/biolreprod.107.062406

[37] Li H, Hung PH, Suarez SS. Ejaculated mouse sperm enter cumulus-oocyte complexes more efficiently in vitro than epididymal sperm. PLoS ONE. 2015;10(5):e0127753. 25996155 10.1371/journal.pone.0127753 PMC4440731

[38] Liu L, Nutter LM, Law N, McKerlie C. Sperm freezing and in vitro fertilization in three substrains of C57BL/6 mice. J Am Assoc Lab Anim Sci. 2009;48(1):39–43. 19245749 PMC2694707

[39] Ramm SA, McDonald L, Hurst JL, Beynon RJ, Stockley P. Comparative proteomics reveals evidence for evolutionary diversification of rodent seminal fluid and its functional significance in sperm competition. Mol Biol Evol. 2009;26(1):189–98. 18931385 10.1093/molbev/msn237

[40] Kawano N, Yoshida M. Semen-coagulating protein, SVS2, in mouse seminal plasma controls sperm fertility. Biol Reprod. 2007;76(3):353–61. 17123940 10.1095/biolreprod.106.056887

[41] Bromfield JJ, Schjenken JE, Chin PY, Care AS, Jasper MJ, Robertson SA. Maternal tract factors contribute to paternal seminal fluid impact on metabolic phenotype in offspring. Proc Natl Acad Sci U S A. 2014;111(6):2200–5. 24469827 10.1073/pnas.1305609111 PMC3926084

[42] McGill TE. Sexual behavior in three inbred strains of mice. Behaviour. 1962;19:341–50. 10.1163/156853962X00087

[43] McGill TE, Blight WC. The sexual behaviour of hybrid male mice compared with the sexual behaviour of males of the inbred parent strains. Animal Behav. 1963;11:480–3. 10.1016/0003-3472(63)90265-3

