# Supplementary material for: A simple method for repeated in vivo sperm collection from laboratory mice
Source: J Assist Reprod Genet. 2024 Jul 17;41(9):2537–46. doi: 10.1007/s10815-024-03201-x (PMC11405545; doi:10.1007/s10815-024-03201-x)
Supplement: Supplementary file 1 — Supplementary file1 (DOCX 15 KB) [file 10815_2024_3201_MOESM1_ESM.docx]

Supplementary Table 1: Number of spermatozoa in 10˄6 /ml of successfully collected ejaculates.

| Male ID | Ejaculation number | | | | | | | | | |
| --- | --- | --- | --- | --- | --- | --- | --- | --- | --- | --- |
|  | 1 | 2 | 3 | 4 | 5 | 6 | 7 | 8 | 9 | 10 |
| 1 |  | 2.1 | 22.0 | 16.0 | 9.4 | 15.2 | 11.2 | 16.1 | 18.0 | 19.2 |
| 2 |  |  | 24.3 | 14.4 | 13.6 | 15.4 | 19.2 | 46.8 | 33.6 | 11.6 |
| 3 |  | 6.0 |  |  | 16.0 | 2.4 |  | 15.2 | 13.8 | 14.0 |
| 4 |  |  |  | 25.3 | 4.0 | 18.0 | 64.8 | 16.4 | 27.2 | 23.6 |
| 5 |  | 15.6 | 4.8 | 17.2 | 21.2 | 16.0 | 2.4 | 18.8 | 21.6 | 0.4 |
| 6 |  | 13.2 | 19.2 | 25.6 |  | 52.8 | 37.2 | 14.0 |  | 20.0 |
| 7 |  | 33.1 | 18.4 | 24.4 | 26.4 | 3.1 | 40.0 | 21.6 |  | 16.8 |
| 8 |  | 7.6 | 12.8 |  | 4.2 | 13.2 | 8.6 | 14.4 | 12.8 | 18.4 |
| 9 | 5.1 | 23.2 | 10.2 | 18.4 | 8.0 | 41.6 | 18.8 | 25.8 | 24.0 | 16.4 |
| 10 |  |  | 7.2 | 14.4 | 12.0 | 13.6 | 7.0 | 29.6 | 26.0 | 48.0 |
